# What Is the Impact of Energy Expenditure on Energy Intake?

**DOI:** 10.3390/nu13103508

**Published:** 2021-10-05

**Authors:** Anja Bosy-Westphal, Franziska A. Hägele, Manfred J. Müller

**Affiliations:** Department of Human Nutrition, Institute of Human Nutrition and Food Science, Christian-Albrechts University Kiel, Düsternbrooker Weg 17, 24105 Kiel, Germany; fhaegele@nutrition.uni-kiel.de (F.A.H.); mmueller@nutrfoodsc.uni-kiel.de (M.J.M.)

**Keywords:** total energy expenditure, energy flux, thermogenesis, appetite control

## Abstract

Coupling energy intake (EI) to increases in energy expenditure (EE) may be adaptively, compensatorily, or maladaptively leading to weight gain. This narrative review examines if functioning of the homeostatic responses depends on the type of physiological perturbations in EE (e.g., due to exercise, sleep, temperature, or growth), or if it is influenced by protein intake, or the extent, duration, timing, and frequency of EE. As different measures to increase EE could convey discrepant neuronal or humoral signals that help to control food intake, the coupling of EI to EE could be tight or loose, which implies that some ways to increase EE may have advantages for body weight regulation. Exercise, physical activity, heat exposure, and a high protein intake favor weight loss, whereas an increase in EE due to cold exposure or sleep loss likely contributes to an overcompensation of EI, especially in vulnerable thrifty phenotypes, as well as under obesogenic environmental conditions, such as energy dense high fat—high carbohydrate diets. Irrespective of the type of EE, transient elevations in the metabolic rate seem to be general risk factors for weight gain, because a subsequent decrease in energy requirement is not compensated by an adequate adaptation of appetite and EI.

## 1. Introduction

There are considerable intra-individual variations in daily energy expenditure (EE) due to, e.g., day-to-day differences in physical activity (PA) [1,2,3] or variances in sleep duration (i.e., on weekdays vs. the weekend) [4,5,6,7]. Biologically, it is plausible that a higher EE should inevitably lead to a compensatory stimulation of food intake. Teleologically, this coupling of energy intake (EI) to changes in EE is important for the maintenance of energy balance, ensuring survival. However, it remains incompletely understood whether the homeostatic responses to EE depend on the type of physiological perturbations in EE (e.g., it may differ between exercise, sleep loss, differences in temperature, or growth). In addition, the EI response to EE may be influenced by the extent, duration, timing, and frequency of changes in EE. Since different measures to increase EE can convey discrepant neuronal or humoral signals that help to control food intake, the coupling of EI to EE could be tight or loose, which implies that different ways to increase EE may have advantages or even disadvantages for body weight control. This narrative review examines the evidence and etiology for discrepant relationships between EE and EI under different conditions of EE.

## 2. Main Part

### 2.1. Effect of Increased EE by EXERCISE on EI

Exercise interventions have led to a mean weight loss between −1.5 and −3.5 kg (or −1.3 to −2.6 kg fat loss), with no differences between aerobic and high-intensity interval training at a similar level of energy expenditure [8]. The amount of weight loss from exercise training alone is, however, usually less than expected due to increases in EI that are at least partially compensatory [9,10]. The inter-individual variance in this compensation is high, with most participants in a controlled exercise intervention losing weight, and some gaining weight [10,11]. Since carbohydrate oxidation during exercise has been positively associated with post-exercise EI (accounting for 37% of the variance in EI), it was suggested that post-exercise compensatory eating serves as an adaptive response to facilitate the restoration of glycogen and, thus, carbohydrate balance [12]. This hypothesis implies that training at high intensities would be less recommendable for weight loss because the relative rates of macronutrient oxidation (i.e., energy partitioning) and glycemic control are differently affected, depending on the type and intensity of exercise [13]. Free fatty acids (FFA) are preferentially oxidized by skeletal muscles during low-intensity exercise, whereas glucose is the preferred fuel source during high-intensity exercise [14]. However, after higher intensity exercise, favorable changes in appetite-regulating hormones are found with a greater suppression of orexigenic signals (for meta-analysis see [15]) and greater stimulation of anorexigenic signals [16]. Others have, however, not found intensity-dependent differences in glucagon-like peptide-1 (GLP-1), perceived appetite or subsequent energy intake [17] and the intensity-dependent suppression of orexigenic acylated ghrelin was questioned in a recent systematic review [18]. These acute effects may be explained by blood flow redistribution from splanchnic circulation to skeletal muscles during exercise-suppressing ghrelin levels [16,18], while cytokine release, changes in plasma glucose and insulin concentrations, sympathetic nervous system (SNS)-activity, and muscle metabolism (lactate production, interleukin-6 secretion, see below) likely mediate changes in the anorexigenic signal peptide tyrosine-tyrosine (PYY) and GLP-1 [16].

Investigating the underlying mechanisms contributing to the variances in compensation of EE by EI is complicated because it requires a defined increase in EE (without the possibility of reduced habitual PA caused by exercise interventions), as well as a careful assessment of the compensation (e.g., by monitoring, ad libitum, EI in the short-term or by quantifying stored energy, by measuring changes in body fat and fat free mass in the long-term). While an increase of EE by exercise usually leads to a negative energy balance [19,20,21] and is, therefore, recommended to improve body weight control, there is no compensatory decline in (i) ad libitum EI in response to short-term reductions in energy expenditure due to inactivity [22,23,24,25]. Moreover, (ii) in the long-term, a decrease in habitual PA leads to weight gain [26,27]. This is in line with observational data, the first COVID-19 lockdown associated with higher inactivity due to seizing of business activities, homeschooling, and curfews, has led to a mean increase of 1.57 kg in body weight (95% CI 1.01 to 2.14) compared to the pre-lockdown period [28]. In addition, it was recently shown in 1754 individuals that 28% of activity-related EE is compensated due to reductions in basal EE during the day, leading to a less negative energy balance than expected when engaging in PA [29].

Exercise can lead to an acute negative energy balance due to absent increases in EI in response to exercise-induced elevations in EE for 2–10 h post-exercise [21]. It was proposed that this effect may be due to exercise-induced changes in appetite hormones that cause a prolonged feeding latency due to suppressed ghrelin levels and increased levels of PYY, GLP-1, and pancreatic polypeptide (PP) [30]. These effects seem to be independent of exercise mode (e.g., running, cycling, walking) but, nevertheless, a more pronounced transient suppression of hunger and levels of acylated ghrelin was seen in studies with exercises demanding higher metabolic and mechanical requirement and, thus, possibly leading to muscle damage and higher muscle loading (e.g., running vs. cycling) [31,32]. In line with this observation, subjects with a lower habitual fitness level seem to be more prone to experience an anorexigenic effect in the initial hours after exercise compared to individuals with a higher fitness level [21].

A negative energy balance caused by exercise interventions may be due to long-term effects (e.g., a more steady and persistent increase in exercise levels) that are shown to reduce hypothalamic inflammation and increase sensitivity to anorexigenic hormones [33]. Chronic exercise also seems to increase total and des-acyl ghrelin levels, which is more pronounced in overweight and obese people and in long-term exercise programs. However, it is speculated that these changes are rather due to weight loss compared to exercise per se, as discussed in a recent review [18]. Short-term exercise interventions (between one day and about two weeks) in untrained subjects may also improve the sensitivity of the physiological satiety signaling system, lead one to adjust macronutrient preferences or food choices, and alter the hedonic response to food [34].

Grannell et al. suggested that no muscle-specific signal is required for reduction in appetite since the association between muscle activity and EI is likely reflective of energy utilization, indicating that it is, overall, EE that determines EI [35]. Therefore, the authors propose that AMP-activated protein kinase (AMPK) activity induced by PA could signal energy utilization to the brain [35]. Hypothalamic AMPK mediates incoming peripheral stimuli and influences the response of arcuate nucleus neurons; thus, playing an important role in central appetite control [36]. It remains, however, uncertain whether distal AMPK activity due to system-wide EE is coupled to central AMPK activity in the hypothalamus [35]. Alternatively, an increase in body temperature (see below), lactate production (leading to lower ghrelin secretion), and elevated FFA, as well as an increase in circulating interleukin-6 (IL-6) secreted by muscle cells may be important mediators of the effects of exercise on perceived hunger and subsequent EI (for review see [16]). After high intensities of running, IL-6 concentrations were positively correlated with GLP-1 and inversely with overall appetite sensation [37]. However, high-intensity exercise, which involves large muscle mass, is needed for relevant IL-6 secretion, whereas during low-intensity PA, there is little to no impact on muscle-induced IL-6 [38]. This suggests that alternative mechanisms exist, linking low-intensity PA and appetite control, explaining a better appetite control in individuals with habitual high PA levels [39,40].

One obvious alternative explanation is a higher stimulation of anorexigenic gastrointestinal peptides, and suppression of orexigenic ghrelin in response to an adequately higher ingestion of food due to higher energy requirements with exercise. This is supported by an increase in GLP-1 levels with increasing energy flux (i.e., a higher EE with correspondingly higher EI) at a concomitant decrease in ghrelin levels [24]. The beneficial effects of exercise on energy balance can be fully prevented under conditions of a high-fat, energy-dense diet [22,41,42]. Likewise, under controlled conditions in a metabolic chamber, we found that a high PA level of 1.76 at low-intensity was needed for spontaneous maintenance of energy balance under ad libitum access to an energy-dense Western diet [24]. In line with the idea that a higher amount of food in the gastrointestinal tract improves satiety, even at a negative energy balance, Thivel et al. found that the energy deficit due to 24-h fasting led to a higher subsequent compensation of EI when compared to a matched energy deficit caused by exercise [43].

### 2.2. Effect of Increased EE by Temperature on EI

In homeothermic organisms, a decrease in ambient temperature results in a higher metabolic rate to meet the increased thermogenic demand. Thus, there is an inverse relationship between ambient temperature and food intake [44]. Interestingly, the thermogenic responses to mild cold exposure and overfeeding were found to be highly correlated and related to norepinephrine levels [45], pointing to a common role of brown adipose tissue (BAT) activity for both types of increases in EE. As a non-sympathetic enhancer of BAT activity, the gut hormone secretin mediates diet induced thermogenesis and, thus, induces satiation [46]. The physiological role of prandial and cold-induced thermogenesis in the control of satiation may therefore be conveyed by the same mechanisms (secretin or SNS-activity both increasing BAT activity and, thus, heat generation that is sensed in the brain). Since EE for active thermal regulation decreases with modern heated thermoneutral environments, seasonal increases in body weight during the winter are mainly explained by a decrease in PA [47].

In healthy normal weight men, 60 h of cold exposure (16 °C in a metabolic chamber) increased EE due to increases in sleeping metabolic rate and diet-induced thermogenesis (DIT), but did not lead to a higher EI during a 24 h period compared to a thermoneutral ambient temperature of 22 °C [48]. The lack of differences in EI between both conditions was, however, likely due to an excess EI at both temperatures (+32% at 22 °C and +34% at 16 °C), with ad libitum feeding in an inactive situation. This voluntary overfeeding likely attenuated the decrease of the core temperature at 16 °C. Interestingly, greater decreases in rectal temperature were correlated with increased food intake (percentage overeating (r^2^ = 0.7; *p* < 0.01)). The increase in EI at 16 °C by an average of 900 kJ (n.s.) tended to exceed the average increase in EE of 500 kJ [48].

In contrast to cold exposure, warm temperature (27 °C vs. 22 °C) reduced EE due to reductions in DIT and activity EE [49]. Again, the corresponding reduction in EI was primarily related to increases in body temperature and only secondary to changes in *EE per se.* Additionally, in healthy men, it was shown that a warm temperature (30 °C vs. 20 °C and 10 °C) during rest led to decreased *ad libitum* EI (−1188 kJ compared to 20 °C and −1243 kJ compared to 10 °C, both *p* = 0.002), coinciding with lower perceived overall appetite between breakfast and lunch [50]. Ambient heat stress of 40 °C (with 25% relative humidity) and 50 °C (with 50% relative humidity) both exceeded the upper limit of the thermoneutral zone and led to a 35% and 48% increase in resting energy expenditure by increases in heart rate, ventilation rate, and sweat rate [51]. The impact of this heat stress induced increase in EE on EI remains unknown.

It is tempting to speculate that the thermogenic effect of exercise might contribute to exercise-induced anorexia. Higher EI in weight reduced obese or sleep deprived subjects (one night of sleep deprivation reduced core temperature and increased heat loss due to a cold stimulus [52]), could also be related to reduced body temperature. In line with this idea, evidence suggests that swimming compared to other types of exercise may increase appetite and EI [53,54]. An orexigenic effect of swimming was identified that may not be explained solely by the EE of muscles [55], but may also be due to increased heat loss with immersion in cool water. Post-exercise food intake was on average 44% higher 45 min after exercise in the cold (20 °C or 68 °F) compared to thermoneutral (32 °C or 89 °F) water conditions [56,57]. During swimming in cold water, core temperature is largely maintained by peripheral vasoconstriction whereas this process ends after exiting the water. Cold blood from the periphery then returns to the core where it causes a delayed decrease in core temperature (so called “after-drop”) that explains shivering after leaving the water [58]. Notably, the typical body fat content of professional swimmers is significantly higher than that of runners or cyclists who expend similar or even smaller amounts of energy in their training [59,60]. Higher body fat of female professional swimmers used to be opposed by prescribed “land training” (running or cycling) in the belief that this might help to lower skinfold thickness. In line with this observation, earlier studies have also shown that swimming may not be as effective as other types of exercise for weight management [61] due to an increased energy compensation after exercise. Accordingly, it was shown that exercise-induced anorexia, in terms of subjective hunger and tympanic temperature, were reduced if exercise was performed during cold exposure (12 °C vs. 24 °C and 36 °C), whereas plasma ghrelin and PYY levels did not vary between temperatures [62]. By contrast, a 45-min treadmill walk at 8 °C vs. 20 °C in individuals who were overweight led to greater acylated ghrelin concentrations and subsequent EI [63]. In summary, heat exposure favors weight loss, whereas an increase in EE due to cold exposure likely contributes to a caloric overconsumption.

### 2.3. Effect of Timing and Frequency of EE on EI

Body weight is not steadily increasing, but may show a higher increase during vulnerable situations caused by perturbations in EE. First, the annual weight gain was found to be higher at younger ages <45 y and steadily decreasing in older ages [64]. It remains, however, unclear if the dynamics of weight gain correlate with the dynamics of decreases in EE during one’s life span [65,66]. There is, however, evidence, that the compensation reaction is affected by aging. It was shown that older (in contrast to younger) men (70.0 ±7.0 vs. 23.7 ±1.1 years) failed to spontaneously regain body weight after weight loss and decrease energy intake after overfeeding [67]. Therefore, it seems that the coupling of energy requirement to energy intake is weaker with age. Second, seasonal variation in body mass was associated with a synchronous variation in PA. Body mass peaked in the cold winter months (about +1/2 kg) when PA was low and decreased until mid-summer when daily PA peaked [47,68]. Third, there was a weekly weight cycle with a higher weight early in the week (Sunday and Monday) and decreasing weight thereafter [69,70]. During weekends, EI was higher [71,72] and PA lower [3,70,73]. Interestingly, a compensation pattern was strongest for those who lost or maintained weight and weakest for those who prospectively gained weight. This may suggest that a high variance in EE (high increase followed by a high decrease) is related to improved body weight control. In line with this hypothesis, healthy and physically active adults were leaner, but also more variable in their day-to-day activities compared to less physically active people [7]. This intra-individual variation in PA was inversely related to BMI or % fat mass. In a subgroup of individuals with a low habitual PA, a higher day-to-day variance in PA was associated with a lower BMI compared to individuals with a more constant level of low PA [7]. These results suggest that a high variance in PA could help to prevent weight gain. The variance in higher-intensity PA may, however, be less protective, because in overweight adults, estimates of moderate-to-vigorous PA were more variable than total PA [2]. However, in contrast to this finding, people with higher habitual PAL (and SD of PAL) had a higher ad libitum EI after a high energy preload [74]. This result shows that a high ET leads to long-term adaptation in perceived energy requirement and, thus, EI. Timing of exercise had no impact on the daily EI or changes in body fat in a 12-week supervised multimodal exercise program performed either in the morning or evening in sedentary overweight and obese subjects [75].

### 2.4. Effect of Sleep Duration on EI

Sleep restriction leads to slightly enhanced energy requirements because of an increase in 24 h-EE by about 5%, which is predominantly explained by the energy cost of extended wakefulness [4,5]. This effect is, however, weakened by reduced spontaneous PA and greater sedentary behavior with sleep loss in some subjects [76]. Since observational studies suggest a link between sleep restriction and obesity in all age groups (for review see [77,78]), the net effect of sleep loss should lead to a positive energy balance. In addition, high habitual sleep variabilities with short sleep durations followed by recovery sleep was related to a higher risk for overweight or obesity [79], or increased EI and snack consumption in adolescents [6]. As a potential mechanism, insufficient sleep is associated with decreases in leptin and increases in the hunger-stimulating hormone ghrelin [80,81]. Appetite regulating hormones do, however, not fully explain why sleep loss contributes to weight gain as overeating occurred despite increases in leptin [4,82] and PYY, and decreased in ghrelin [4]. Alternatively, excess energy intake associated with insufficient sleep seems to be mainly explained by hedonic or rewarding effects of food rather than hormonal factors [83]. Recovery sleep after sleep loss led to decreased energy intake (especially of rewarding fat- and carbohydrate-rich foods) and, thus, a slight weight loss [4].

### 2.5. Effect of Energy Requirement Due to Growth, Reproduction, and Changes in Body Composition on EI

Higher energy requirements due to growth, reproduction, or changes in body mass and composition also trigger a higher EI that may be adequate to match an equal energy balance, or maladaptive, leading to weight gain, especially in an obesogenic environment with continuous access to energy dense, highly palatable, ready-to-eat food. Lean body mass and resting metabolic rate were proposed to constitute a biological drive to eat, whereas fat mass was inversely associated with food intake, especially in leaner subjects [84,85,86,87]. In 1993, it was proposed that “the impetus for lean tissue growth, or protein accretion (…) regulates nutrient supply” [88]. In adults with overweight and obesity, total energy demand, reflected by higher spontaneous PA and 24 h-EE, might be more important than fat-free mass (FFM) for determining ad libitum overeating, because 24 h-EE in a metabolic chamber was shown to explain 80% of the relationship between FFM and subsequent ad libitum food intake over 3 days [89]. In this study, higher 24 h-respiratory quotient (RQ), indicating a higher rate of carbohydrate oxidation, was also independently associated with a higher food intake [89]. This finding is in line with previous studies that found that the 24 h-carbohydrate oxidation during energy balance in a metabolic chamber was positively associated and carbohydrate balance was inversely associated with subsequent 3d ad libitum food intake [90] and weight gain [91]. As mentioned above, carbohydrate oxidation during exercise was also positively associated with post-exercise EI (accounting for 37% of the variance in EI) [12]. Interestingly, individuals with habitual nighttime eating behavior also had a higher 24 h-RQ during controlled feeding in a metabolic chamber, which was associated with subsequently increased food intake [92]. This study indicates that nighttime eating behavior may be a consequence rather than a cause of a higher 24 h-RQ. Due to the gluco- or glycogenostatic hypothesis of body weight regulation, characteristics of carbohydrate metabolism (high turnover rate, limited storage, immediate, and tight regulation, and critical role as a fuel source for the central nervous system) explain why an enhanced reliance on carbohydrate oxidation may stimulate appetite to restore blood glucose levels or carbohydrate balance [93,94].

Conversely, after a glycogen-depleting exercise the day before, a higher increase in postprandial carbohydrate oxidation was associated with a higher decrease in hunger ratings [95], supporting a previous observation that higher postprandial glucose oxidation leads to decreased hunger [96]. Likewise, inhibition of intracellular glucose utilization by 2-deoxy-d-glucose has been shown to stimulate hunger and food intake in humans [97]. In 1953, Mayer suggested that decreases in glucose utilization rates were associated with increased hunger [93]. Alternative to the glycogenostatic-hypothesis of appetite regulation [93,94], impaired glucose utilization may be a consequence of central insulin resistance leading to impaired appetite control (for review see [98]). Impaired mitochondrial function was associated with impaired metabolic flexibility and a higher RQ during the night in healthy people with a family history of type 2 diabetes [99], or in patients with type 2 diabetes [100]. Although a higher RQ is a sensitive marker of a positive energy balance, and increases with a high glycemic load diet, the previously mentioned protocols used a carefully controlled energy and macronutrient intake and, thus, reveal metabolic characteristics of the participants rather than a response to diet.

When an increase in EE cannot be maintained and the energy requirement decreases, energy balance and body fat content may increase, e.g., after detraining in competitive athletes [26,27], with aging [101], due to the age-related loss in skeletal muscle mass and high metabolically active organ masses per kg lean mass [102] or PA. In addition, further evidence for a persistent adaptation of appetite to a high EE comes from the increasing risk of weight gain after each pregnancy [103]. Higher EE during pregnancy, with a sudden decrease in energy turnover after delivery (especially without breastfeeding), may therefore increase the risk of overweight or obesity. A transient disproportionate high increase in lean compared to fat mass during a growth spurt may also explain the increased risk of obesity in children with a high birth weight [104], a high-protein intake during the first 2 years of life [105] or early BMI rebound [87,106], as well as precocious puberty [107]. Finally, as high body mass EE is higher in people with obesity and may, thus, impede weight maintenance after successful weight loss is tantamount to a decrease in EE [108]. Higher compensatory EI, as a result of the lower EE, has been elegantly calculated from the difference between actual and predicted weight loss following therapy with sodium-glucose cotransporter 2 inhibitors that lead to unnoticeable “energy loss” via glucosuria [109]. The increase in EI above baseline was about 95 kcal/d for every kg of weight loss.

### 2.6. Effect of Dietary Protein on EE and EI

There is a U-shaped association between dietary protein concentration and heat production [110]. While the specific dynamic action of proteins is well known, the increased thermogenesis in response to overfeeding an unbalanced, severely low protein diet (i.e., 2–3% energy in protein compared with protein restricted, 5–8%, normal, 10–20%, and high protein diets, and >25% protein energy, respectively) was suggested to regulate the metabolic supply of essential nutrients by dissipating some of the excess energy (i.e., ‘homeostatic waste’, according to Max Kleiber) [110]. However, in the long-term, a very low protein diet, as well as diets devoid of at least one essential amino acid, result in a loss of lean body mass, malnutrition, anorexia, and hunger suppression. The U-shaped association between protein intake and energy expenditure suggests that the costs of weight gain (as expressed as % metabolizable protein energy) are increased at low as well as high protein intake.

At a low protein intake, protein requirements drive food intake, resulting in hyperphagia, a specific appetite for protein and an increase in fat mass [111]. In regard to overfeeding a low protein diet, increased thermogenesis is explained by elevated triiodothyronine levels, an enhanced thermogenic response to norepinephrine, and an increase in the secretion of fibroblast growth factor 21 (FGF21). FGF21 is considered a hormonal mediator that causes browning, thus, activating thermogenesis [112,113].

Since, in healthy subjects, there are considerable interindividual variances in the metabolic response to overfeeding, different metabolic phenotypes have been proposed (for reviews, see [113,114]). The susceptibility to gain weight has been related to low REE, PA, and SNS activity, where acute overfeeding for 24 or 48 h at 200% of one’s daily energy needs of a severely low protein diet (i.e., an acute disruption of energy and protein balance) may serve as a ‘stress test’ to identify this phenotype. Then, the increase in the plasma levels of FGF21 in response to severely low protein overfeeding may serve as a biomarker of an elevated risk of weight gain [115]. Following this specific protocol, subjects with low increases in 24 h-EE and plasma FGF21 concentrations are considered ‘thrifty’ (or ‘metabolic efficient’) phenotypes and gain the most weight during the follow-up of overfeeding while they save energy in response to caloric restriction [114,116]. By contrast, a ‘spendthrift phenotype’ markedly increases EE and FGF21 in response to severely low protein overfeeding and, therefore gains less weight when compared to a ‘thrifty phenotype’. Thus, a ‘spendthrift phenotype’ seems to be protected against being overweight [114,116]. When compared with ‘spendthrift’ individuals, ‘thrifty’ subjects also showed smaller increases of EE in response to high protein overfeeding [117]. Increases in EE in response to a hypercaloric severely low protein diet are explained by an increased DIT, energetic costs of muscle activity, and thermogenesis due to non-exercise activities (NEAT) [114]. However, it is unclear, whether high EE results from hyperphagia or vice-versa.

In contrast to fat overfeeding in normal weight subjects, a controlled 8-week overfeeding with low, normal, and high amounts of protein (i.e., up to 3.0 g protein/kg body weight x d corresponding to 25% of energy from proteins compared with the required daily protein intake of 0.8–1.2 g/kg body weight x d or 10–15% percentage energy) at increasing EI (up to 3439 kcal/d) in a respiratory chamber increased body weight, nitrogen retention, EE (24 h-EE, sleeping EE), body and muscle protein synthesis, as well as protein oxidation, where the latter finding reflects that amino acid supply had exceeded protein deposition [118]. Over the study period, weight gain was 3.2, 6.1, and 6.5 kg in response to overfeeding at 5, 15, and 25% protein, respectively [113]. The relatively moderate weight gain observed in the low protein group resembles energy dissipation in response to overfeeding. Accordingly, increases in EE correlated with protein rather than energy intake [118].

In addition to long-term adaptations to changes in protein intake, there is an immediate response to the intake of proteins in meals. After isocaloric test meals, dietary proteins stimulate DIT and decrease EI, with different protein sources differing in their metabolic effects [119,120]. As for individual macronutrients, DIT is about 0–3% for fat, 5–10% for carbohydrates, 20–30% for proteins, and 10–30% for alcohol, with a mean of 10% of total EI in response to a mixed meal [121,122]. After isocaloric test meals with 50% protein, the thermic effect of whey, casein, and soy proteins (as calculated as the incremental increase in energy expenditure in response to the different test meals above baseline) differed between 11.6 and 14.4%, exceeding the responses to carbohydrate intake by 4.9 to 7.7% [120].

The energy costs of digesting, absorbing, and metabolizing proteins is about 23% of their energy content. From the point of view of energy metabolism, there is a difference between the gross energy value of protein and its metabolizable energy (i.e., 5.25–5.98 vs. 3.11 kcal/g; [121]). While acute effects of high protein intake relate to amino acid oxidation, urea production, and decreases in protein degradation, long-term adaptations to a high protein intake increase protein turnover (i.e., the sum of protein synthesis and protein degradation). The daily rate of protein turnover is 300 to 400 g, which exceeds protein intake of 60 to 80 g/d, reflecting that the reutilization of endogenous amino acids from the breakdown of body protein is higher than the intake of dietary protein and amino acids [122]. Major mechanisms explaining protein-induced thermogenesis relate to energy-consuming metabolic processes, i.e., ureagenesis, gluconeogenesis, and protein synthesis. About 20% of the energy content of amino acids is required for ureagenesis [123]. Most urea is excreted in the urine as a function of dietary protein intake. Some urea is lost in the sweat and the gut. After microbial hydrolysis in the colon, urea recycles as nitrogen and can be retransformed into liver proteins [123]. Physiologically, there is a continuous synthesis and breakdown of proteins. Protein turnover per day involves 3–4% of whole body protein with the gut, liver, and skeletal muscle as major contributors; it accounts for about 20% of REE, depending on the amount of muscle mass [122]. After a meal, the increase in EE is positively correlated with plasma amino acid concentrations, protein synthesis, and energy-consuming pathway rates, i.e., ureagenesis and gluconeogenesis.

Proteins are more satiating than carbohydrates and fats, whereas the effects of proteins are dose-dependent [124]. The anorectic effect of proteins on EI have been related to slower gastric emptying, changes in plasma incretins (GLP1, PYY, ghrelin, cholecystokinin), protein synthesis, amino acids, and/or increased energy expenditure targeting different brain areas involved in the control of appetite, hunger, and satiety (i.e., the mediobasal hypothalamus, the hindbrain, and the limbic area). Protein-induced satiety is associated with protein-induced increases in EE. In addition, “high protein—high fat—low carbohydrate diets” increase ketogenesis, where elevated plasma ketone bodies result in a more anorectic effect when compared with “high protein—normal fat—normal carbohydrate diets” [120]. High protein diets with a high proportion of ketogenic amino acids (i.e., leucine and lysin) result in increased satiety [124].

At a low protein intake, there seems to be a biological control of the intake of protein and essential amino acids. By contrast, at a sufficient (i.e., a normal and a high) protein intake, there is no biological control of protein intake and food intake is then driven by energy needs, conditioning, and reward-driven mechanisms [124]. At a high protein intake, the plasma concentrations of ghrelin and neuropeptide Y are low, while plasma leptin levels are high [122] altogether resulting in feelings of satiety and fullness. In humans, at least 30 g protein per meal are needed to result in a measurable anorectic effect [111]. The protein-satiating effect is observed in response to single meals as well as for high protein-ad libitum diets lasting for months. High protein diets are successfully used in obese subjects for either weight and fat mass losses (as relatively high protein-hypocaloric diets) and during weight maintenance after weight loss (as high protein ad libitum diets) [125].

Taken together, there is a U-shaped association between protein intake and EE, which increases at low as well as at high protein availability. In addition, there is an inverse association between protein intake and EI, with a high EI at low protein intake and a low EI at high protein intake. It becomes evident, that dietary protein differently counterbalances EE and EI, depending on its concentration, which, thus, differently influences energy balance. Vice versa, energy balance impacts protein utilization. However, acute changes (e.g., in response to overfeeding a severely low protein diet or after isocaloric test meals with different protein sources) need to be differentiated from long-term effects of high protein diets, as used in obese patients for weight maintenance after weight loss.

## 3. Conclusions

In conclusion, an increase in EE leads to increases in EI that may be adaptive and compensatory, or maladaptive, leading to weight gain depending on the type of EE. This implies that not only EE per se, but also neuro-endocrine or metabolic effects that signal an increased energy demand under conditions of PA, cold exposure, sleep loss, a very low (or very high) protein diet, or growth and reproduction, are involved in the control of EI. Higher exercise, or PA and heat exposure favor weight loss, whereas an increase in EE due to cold exposure or sleep loss likely contributes to an overcompensation of EI, especially in vulnerable individuals with unfavorable energy partitioning (higher propensity for carbohydrates instead of fat oxidation), as well as under obesogenic conditions, such as an energy dense diet (for summary see Table 1). Irrespective of the type of EE, transient elevations in metabolic rate seem to be general risk factors for weight gain because a decrease in energy requirements is not compensated by an adequate adaptation of appetite and EI. The impact of day-to-day variance and timing (circadian variation) in energy demand due to PA, cold exposure, sleep loss, or protein intake on body weight control needs to be further investigated using the setting of a metabolic chamber.

## Figures and Tables

**Table 1:** Impact of different types of energy expenditure on energy intake and energy balance.

	EE	EI	EB
high protein intake	↑	↓	↓
high habitual PAL	↑	↓(↑)	↓
cold exposure	↑	↑	↑
heat stress	↑	↓	↓

EB, energy balance; EE, energy expenditure; EI, energy intake; PAL, physical activity level.
